# dCA1 cells encode and update contextual place preference

**DOI:** 10.3389/fnsys.2026.1702298

**Published:** 2026-04-09

**Authors:** Kwon Choi, Ignitius Ezekiel Lim, Ajn Vats, Sonni Marie Tarver, Tashonda Benoit Vaughn, Olalekan Michael Ogundele

**Affiliations:** Department of Comparative Biomedical Sciences, School of Veterinary Medicine, Louisiana State University, Baton Rouge, LA, United States

**Keywords:** absolute, spatiotemporal, firing rate, magnitude, reward

## Abstract

Pyramidal cells in the dorsal hippocampus (dCA1) are excitatory neurons modulated by environmental cues. While a population of dCA1 cells encodes spatial location, other groups are activated by reward probability and encounters. *Since “rewards” are predicted at “locations,” we sought to determine how spatiotemporal coding patterns in the dCA1 resolve contextual preference and subsequent change in preference that is driven by reward encounters.* Specifically, we examined these encoding patterns in biased place-preference tasks for simple reward acquisition and for complex discrimination of reward magnitudes. Initial behavioral tests for mice without neural implants revealed a higher sensitivity to discriminating between *two locations* associated with reward magnitude, in comparison to reward detection. Analysis of dCA1 single-unit spatiotemporal activity during pre-conditioning revealed that these cells exhibited peak firing as they approached the less preferred context. Therefore, when the contextual preference is biased toward a reward or a higher-magnitude reward, a change in dCA1 firing rate around context entry events reflects the updated spatial preference. Interestingly, the context of lower preference with no associated reward or a lower-value reward elicits a stronger firing response than the alternative contexts with higher reward values. Together, we conclude that the spatiotemporal firing patterns of dCA1 single units and the threshold of peak FR change encode contextual preference. Ultimately, the spatiotemporal pattern is updated (remapped) when there is a change in the contextual preference driven by reward contingencies.

## Introduction

Neural circuits are central to adaptive behavioral mechanisms that govern survivability in an organism’s natural habitat (Tovote et al., 2015; Plas et al., 2024; Park et al., 2021; Zhou et al., 2017). These circuits cut across the mesocorticolimbic and forebrain centers and underlie innate defensive behavior (Zhao et al., 2023; Carli and Farabollini, 2022), motivated feeding/foraging (Sternson et al., 2013; Hao et al., 2020), stress response/fear, reproductive behavior, and context (valence) discrimination (Adams et al., 2012; Fischer and O’Connell, 2017; Rapp and Nawrot, 2020; Kassraian et al., 2023). Reciprocal connections between the mesocorticolimbic and forebrain circuits detect environmental cues about reward and aversion, and facilitate their recall to guide future decision-making (Cui et al., 2023; Castillo Díaz et al., 2022; Dalenberg et al., 2018; Coley et al., 2021). Adaptive learning is evident in place preference/aversion tasks, where animals show an affinity for locations, cues, or events generally associated with a reward and tend to avoid aversive places or cues (Napier et al., 2013; Roux et al., 2003). To recapitulate complex environmental scenarios, previous studies have shown that animals discriminate between cues or locations based on a mixed probability of getting rewarded with punishment (Anderson, 2019; Orsini and Simon, 2020).

The hippocampus encodes information about space, location, and context. The dorsal hippocampus (dCA1) plays a crucial role in working memory and facilitates the computation of novelty for long-term storage (Dimsdale-Zucker et al., 2022; Liu et al., 2022; McKenzie and Buzsaki, 2016; Russo et al., 2024). The role of the hippocampus in novelty detection stems from its mechanism of comparing new information entries in working memory with previously stored cortical memory (Lisman, 1989, 1999; Lisman and Grace, 2005). Since the environment is enriched with novel sensory stimuli, weight and valence assignments are bolstered by contextual and temporal relevance (Barbeau et al., 2017; Frank and Kafkas, 2021; Gómez-Ocádiz et al., 2022; Valenti et al., 2018). Novel stimuli that directly relate to survival, adaptive behavior, rewards, and punishment are prioritized for long cortical storage and guide future behavioral response (Braun et al., 2018; Cowan et al., 2024; Kalbe and Schwabe, 2022).

In addition to the well-studied spatial coding place cells, a population of dCA1 neurons that predict or detect rewards along the exploration path has been described (Gauthier and Tank, 2018; Jarzebowski et al., 2022). Since spatial novelty can be rewarding, there is a rationale to test whether spatiotemporal mapping of dCA1 reward-sensing ensembles can predict spatial preference and a change in spatial preference that is driven by reward contingencies. Based on this concept, the current study proposes that the spatiotemporal maps of dCA1 ensembles can predict contextual behavioral preference, and that these maps are updated to reflect a change in the preferred context. To test this concept, a sucrose conditioned place preference (sCPP) task was modified such that the initial preconditioning phase depicts the animal’s initial spatial preference, and subsequent sucrose reward bias was aimed at driving a change in the spatial preference. Therefore, sampling spatiotemporal peak firing rates of dCA1 ensembles during the preconditioning and test phases provides a premise for assessing ensemble dynamics relative to spatial preference and a change in preference driven by an absolute or a magnitude reward comparison.

Our results showed that dCA1 neurons were more responsive to reward probabilities than reward encounters. For these tasks, entry events into less preferred contexts, reward omissions, or rewards of lower value elicited stronger responses than preferred context entry, reward encounters, or encounters of reward with a higher value. The spatiotemporal firing pattern of dCA1 putative units encodes the initial pre-conditioning contextual preference and reflects a preference change when the sCPP task is biased with reward modalities. Spatiotemporal dCA1 putative unit population dynamics also showed that magnitude discrimination tasks produce stronger memory traces than simple rewards detection tasks. As such, when rewards were omitted during the test sessions, the change in peak FR for dCA1 neurons was significantly greater than the change during reward encounters in the conditioning phase. Conversely, for reward-detection tasks, the change in firing rate was comparable during conditioning reward encounters and during test reward omission.

## Materials and methods

### Experimental animals

Two separate groups of mice were used for the experiments. In the first cohort (*n* = 20), mice without neural implants were used to assess the behavioral response to absolute and magnitude sCPP task modalities. In the second cohort (*n* = 7), mice had neural implants and were used to evaluate spatiotemporal coding during sCPP tasks. The IACUC committee at Louisiana State University approved all procedures. Mice were kept in standard laboratory housing with alternating light/dark cycles (12:12 h). Food and water were provided *ad libitum.* Mice without implants were housed in groups of 5 per cage (same sex). Animals with neural electrode implants were housed individually and allowed to socialize in controlled laboratory settings.

### Sucrose conditioned place preference (sCPP)

**FIGURE 1:**
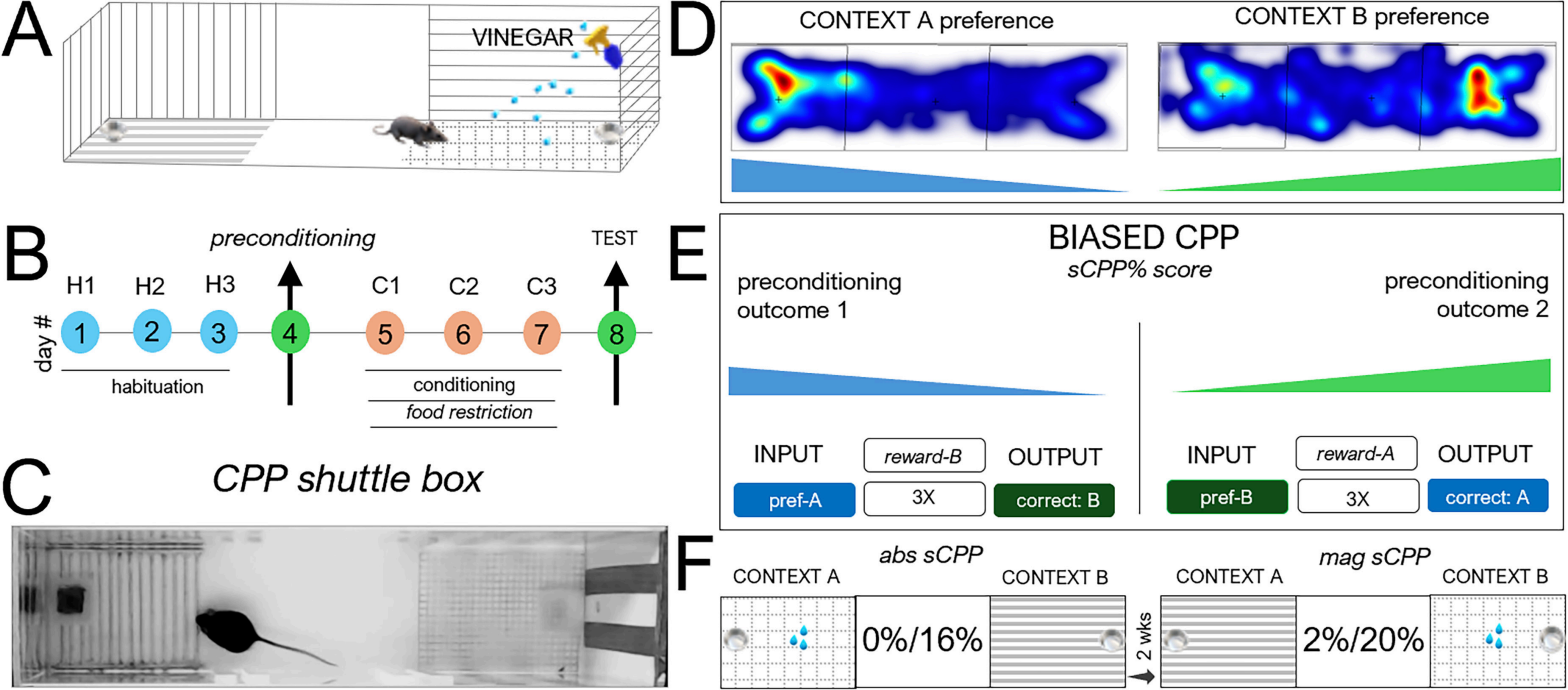
Biased sucrose conditioned place preference (sCPP). **(A)** Schematic illustration of a shuttle box designed for sCPP tasks. Contexts were distinguished spatially (left/right) and by three sensory cues. (i) visually distinct wall patterns, (ii) grid or horizontal floor bars to create distinguishable tactile patterns, and (iii) light vinegar spray in one context for odor discrimination. **(B)** Experimental timeline for habituation (3 sessions), preconditioning (1 session), conditioning (3 sessions), and test (1 session) phases. **(C)** Representative picture of a mouse in a CPP test chamber showing visual/tactile cues and location of sucrose rewards pots. **(D)** Representative preconditioning heat maps for two mice with *context A* or *context B* preference, respectively. **(E)** Schematic illustration of biased sCPP task execution for mice with preconditioning context A or B preference. **(F)** Schematic illustration of biased reward conditioning for absolute (0%:16% sucrose) and magnitude (2%:20% sucrose) sCPP tasks.

Adult C57BL/6 mice (*n* = 20 mice, 10M/10F, 12–16 weeks) weighing 25–30 g *(no neural implant)* were used for sCPP behavioral tests. A biased sucrose CPP test was used for this experiment. A shuttle box with dimensions 40 cm (L), 10 cm (W), and 12 cm (D) was divided into three sections. The central area was the starting zone, separated from the two edge compartments by removable dividers. The two edge compartments were used as target contexts and were distinguishable by three sensory (visual, tactile, and olfactory) cues (Figure 1A). Each session was 10 min and was performed on the same day for all mice.

#### Absolute sCPP

This is a reward detection task in which one context had a reward (sucrose) during the conditioning phase. For this task, context A had vertical lines on all three walls, no odorant, and a grid mesh floor. Conversely, Context B had horizontal lines on all walls, 10% vinegar sprayed lightly, and horizontal bar floors.

#### Magnitude sCPP

This is a discrimination task in which mice explored two rewards of different magnitudes during the conditioning phase. Here, the context was changed for novelty with a different mix of floor (tactile), wall (visual), and vinegar (olfactory) locations.

Mice were habituated to the testing chamber at 72, 48, and 24 h (H1–H3). Subsequently, mice were assessed in a preconditioning task to determine the spatially preferred and non-preferred contexts per mouse (Figures 1B,C). This output was determined by the comparison of the total time spent in each context during the preconditioning session. Once the initial context preference was determined per mouse, three biased conditioning sessions were performed (daily) such that a reward (16% sucrose, *abs sCPP*) or a reward of higher value (20% sucrose, *mag* sCPP) was dispensed at the preconditioning non-preferred context. Before daily conditioning sessions, food restriction was performed for 8–10 h (Figure 1B). Based on the preconditioning output, the non-preferred context was set as “target,” and the preferred context was set as “non-target” for the subsequent conditioning and test phases. Representative heatmaps of preconditioning outcomes for two mice, in which one mouse preferred context A and the other showed a preference for context B are shown in Figure 1D. Contextual preference during the preconditioning phase was determined by a greater than 55%-time distribution in one of the two contexts. For contexts A or B, PrC preference, a biased reward, or reward magnitude presentation was performed during the conditioning phase (Figure 1E). For *absolute sCPP* conditioning sessions (C1–C3), 16% sucrose was dispensed into the reward pot in the target context (PrC, *non-preferred*), and no reward was delivered in the non-target context (PrC, *preferred*) (Figure 1F). For *mag sCPP* conditioning (C1–C3), both contexts contained a reward. However, the target context (PrC, *non-preferred*) contained 20% sucrose while the non-target context (PrC, *preferred*) contained 2% sucrose (Figure 1F). Subsequently, in the test session, animals were tested without a reward present. The sCPP score was calculated as the percentage of time spent on the target side relative to the total time spent on both sides.


Ttarget/(Tnon-target+T)target×100

### Neural electrode implant and spatiotemporal analysis of dCA1 ensembles

**FIGURE 2:**
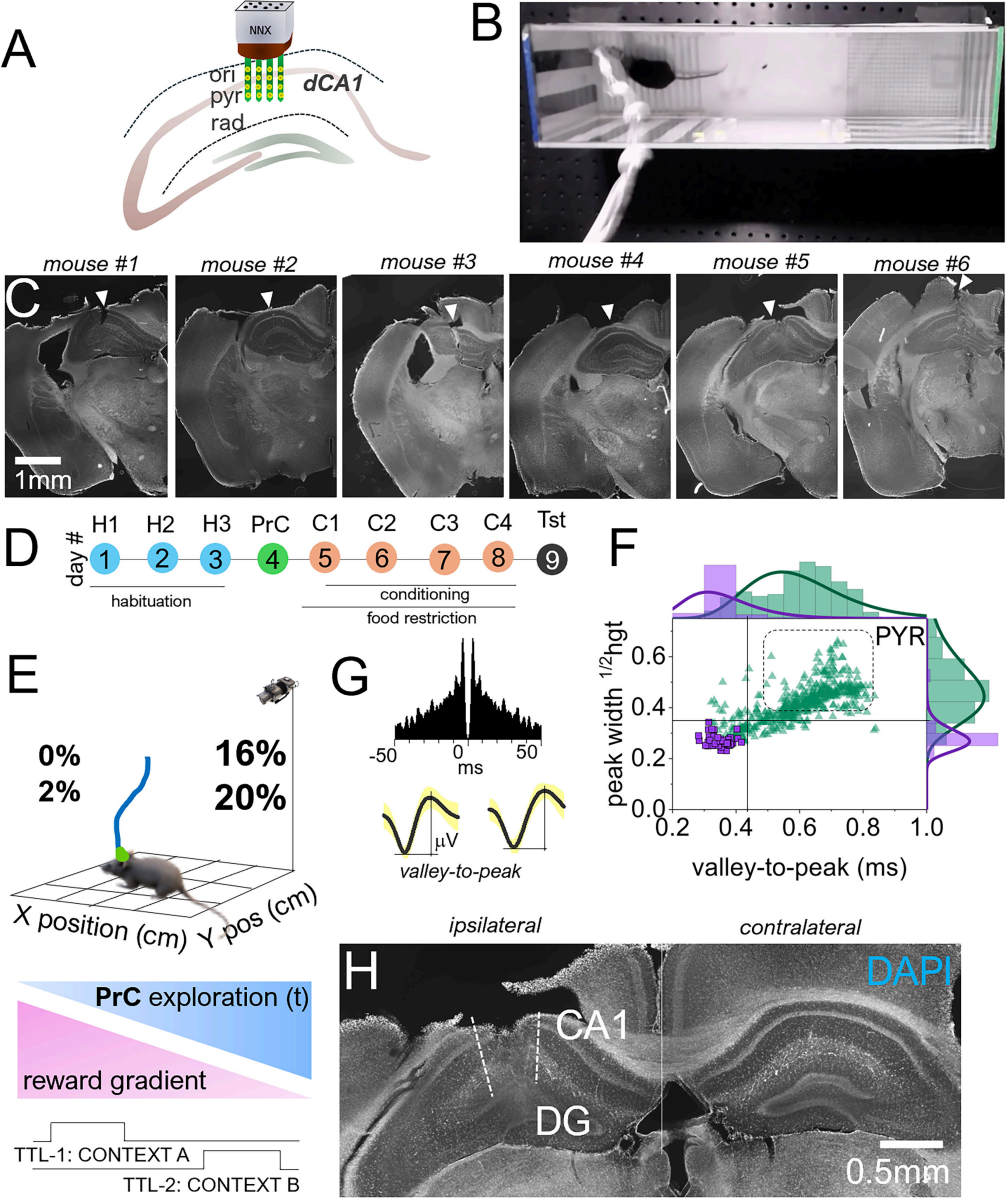
sCPP tasks with dCA1 single unit sampling. **(A)** Schematic illustration of a neural probe implant (16-channel electrode, 4 × 4) in the dCA1. **(B)** Representative picture showing a mouse with neural implants and a tether in a shuttle box during an sCPP task. **(C)** Fluorescence (DAPI) images showing neural electrode implant sites (scale bar 1 mm). **(D)** Timeline for neural recording and sCPP task sessions in mice with neural implants. Note that mice with neural implants performed four conditioning sessions. **(E)** Schematic depicting spatiotemporal mapping of dCA1 pyramidal single unit activity relative to the mouse XY position (cm) in the shuttle box. **(F)** Edge histograms and cluster plots of putative dCA1 pyramidal cells based on waveform valley-to-peak duration and peak width at half height. **(G)** Representative waveforms and autocorrelogram of putative dCA1 pyramidal cells. **(H)** Representative DAPI fluorescence image showing tissue scar of recovered neural electrode shanks in the dCA1 (scale bar 0.5 mm).

Adult male *Vglut2^Cre^* mice (*n* = 7) weighing 25–30 g were used for this study. Animals were anesthetized with ketamine/xylazine (100/10 mg/kg). Once the plane of anesthesia was established, a toe pinch was performed to verify the absence of sensation. The head was fixed on a stereotaxic frame. Sterile surgical preparation techniques were used. These included shaving and cleaning the scalp area with iterations of iodine and alcohol solutions. A local anesthetic agent was applied before an incision was made to expose the skull surface. By locating the bregma, anterior-posterior (AP: −2.18 mm) and medial-lateral (ML: 1.0–2.0 mm) coordinates that corresponded to the dorsal CA1 were determined and marked. A drill tool was used to prepare a 2.5 mm craniotomy over this point to expose the dura. A sharp, bent, clean needle was then used to remove the dura. Sterile saline was applied over the craniotomy to prevent dryness. A second craniotomy housed a screw to which the ground and reference wires were connected. Once the ground and reference wires were in place, 16-channel silicon probes (Neuronexus, United States), were gently lowered into the dCA1 such that electrode contact sites were in the cell body layer (DV: 1.3–1.5 mm) (Figure 2A). The probe was tethered to a head stage to detect the spontaneous activity of putative pyramidal cells (0.3–5 kHz). The anatomical location of the probe was micro-adjusted to detect robust signals on electrode contact sites in the DV range. Once the final location was set, dental acrylic was applied to cast the implant onto the skull. Seven days after surgery (recovery), animals began behavioral testing (Figure 2B). The impedance of electrode contact sites measured between 0.8 and 2.0 MΩ. The location of the neural electrodes in the dCA1 was validated *post hoc* using histology (Figure 2C).

### Synchronized dCA1 recording and CPP task

Dorsal CA1 spikes were sampled during the preconditioning (PrC), conditioning (C1, C2, C3, and C4), and test (Tst) phases (Figure 2D). Implanted neural electrodes were tethered to a preamplifier head stage and recording controller (Intantech, United States). Through a Noldus Mini I/O box, TTL signals triggered by zone entry and exit were transmitted to the digital-IN channels of the recording controller by wired connections (Figure 2E). All behavioral tasks were acquired with Ethovision XT17 software, which uses a machine-learning algorithm for stable rodent tracking. Body point tracking was used to determine when the animal crossed into a zone. Behavior-driven TTL signals were used in time-locking zone entry events with continuously sampled dCA1 spikes.

### Spike sorting and single-unit detection

In an Offline spike sorter (OFSS, Plexon, United States), extracellular spikes were pre-processed with a Butterworth filter (300–5,000 Hz) to remove anatomical drifts and local field potential artifacts. Single-unit clustering of the recorded spikes was performed by principal component analysis (PCA) to detect putative dCA1 principal neurons. An amplitude discrimination step was implemented in the OFSS to improve the signal-to-noise ratio. A lower peak crossing threshold that is 5x the root mean square (RMS) was set for each electrode channel to eliminate noise and artifact spikes (Quiroga, 2012; Swindale et al., 2017; Chung et al., 2017). Unsorted spikes were manually invalidated where necessary in OFSS. Since more than one putative unit was detected on each electrode channel, spikes were further discriminated against based on the interspike interval (Chung et al., 2017; Quiroga, 2012).

### Characterizing putative pyramidal units

Waveforms of clustered units were inspected across all channels. Viable units were accepted based on the autocorrelogram (ACG), firing rate (FR, Hz), and waveform valley-to-peak time (μsec) (Figure 2F). Putative units with a valley-to-peak time range of 400–800 μs and distinct ACG peaks at 0 ms, followed by a rapid decay (50 ms), were characterized as putative dCA1 pyramidal cells (Sotres-Bayon et al., 2012; Barthó et al., 2004). Interneuron ACGs have a distinct trough at 0 ms, with sustained activity. Interneurons were further distinguished from pyramidal cells by the absence of complex spiking and higher firing rate scores and were excluded from subsequent analysis. Putative units identified as principal cells had a mean firing rate of <5 Hz (Figure 2G). Based on the shape of the waveform, ACG, and firing rate, we expect that most of these putative neurons are glutamatergic cells (Moorman and Aston-Jones, 2015; Barthó et al., 2004).

### Histology and verification of dCA1 recording sites

After CPP behavioral tasks with neural recording, mice were euthanized by isoflurane exposure in a desiccator. The concentration of isoflurane was increased until the animal became unconscious, and death was confirmed by the absence of breathing and toe pinch response. Transcardial perfusion with 4% paraformaldehyde (PFA) in PBS was performed subsequently. The brain was preserved in 4% PFA-PBS and was cryopreserved in the same (fresh) solution containing 30% sucrose. A total of 50 μm cryostat sections containing the dorsal hippocampus were mounted on slides and stained with DAPI to visualize the dCA1 tissue scaring site that indicates the anatomical location of the recording electrode shanks (Figure 2H). Fluorescence images were obtained using a Ni-U fluorescence upright microscope (Nikon AR software) equipped with a Moments camera (Teledyne Canada).

### Statistical analysis

#### Sucrose conditioned place preference tests (*n* = 20 mice, 10 M/10 F)

Statistical analysis was performed using Origin Pro 2024 software (Origin Labs, United States). *Biased sCPP* scores were compared for the preconditioning and test phases using a paired *T*-test for males, females, and all mice (male and female) (*n* = 20). Outcomes for the preconditioning (PrC), conditioning (C1, C2, C3), and test (Tst) sessions were compared using One-Way ANOVA (repeated measures) with *Dunnett’s post-hoc test*. Comparison of absolute and magnitude sCPP outcomes was done using a paired *T*-test.

#### Sucrose conditioned place preference task with dCA1 recording (abs sCPP: *n* = 7, mag sCPP: *n* = 6)

Sucrose conditioned place preference outcomes for mice with neural implants were compared across experimental sessions (PrC, C1, C2, C3, C4, Tst). Sorted spikes for sampled putative dCA1 principal cells were exported to Neuroexplorer (Nex Technologies, United States) for further analyses. Results from spike analysis and Ethovision XT17 behavioral tracking were further analyzed in OriginPro 2024 software. Normality distribution was determined using the *Kolmogorov-Smirnov* test. Comparison of dCA1 ensemble activity across experimental sessions was determined using One-Way ANOVA (repeated measures) with *Dunnett’s post-hoc test*. Change in firing rate of dCA1 neurons during context entry events in the pre-conditioning and test phases was compared using Student’s *T*-test.

## Results

### Absolute sCPP (*n* = 20, 10 M/10 F, *no neural implant*)

**FIGURE 3:**
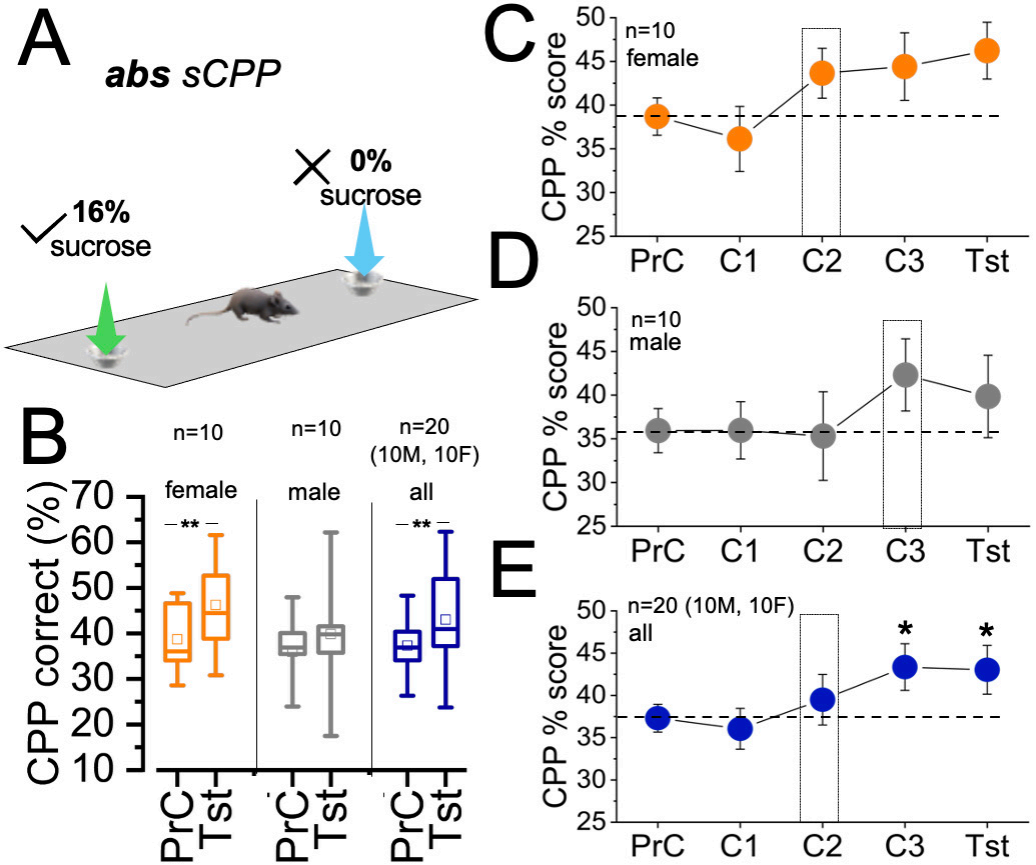
sCPP% score for absolute sCPP conditioned with 0%:16% sucrose. **(A)** Schematic illustration of absolute(abs) sCPP with biased reward conditioning. **(B)** Composite graph comparing preconditioning and test sCPP% scores for female (*t* = –3.47, DF = 9, *p* = 0.007), male (*t* = –1.27, DF = 9, *p* = 0.24), and male/female (*t* = –3.04, DF = 19, *p* = 0.007) cohorts (paired *T*-test). **(C)** Interval plot comparing female sCPP% scores across sessions (One-way ANOVA, OWA, Dunnett’s *post-hoc* test, F = 262.45, *p* < 0.0001). **(D)** Interval plot comparing male sCPP% scores across sessions (OWA, Dunnett’s *post-hoc* test, F = 111.02, *p* < 0.0001). **(E)** Interval plot comparing male/female (all) sCPP% scores across sessions (OWA, Dunnett’s *post-hoc* test, F = 295.25, *p* < 0.0001| versus PrC, C3: *p* = 0.02 and Tst: *p* = 0.03). *p < 0.05, **p < 0.01.

For biased sCPP tasks, the non-preferred (nP) side during preconditioning (PrC) was set as the target (Tg), and the preferred side (P) was the non-target (nTg). In the following three conditioning sessions, mice obtained 16% sucrose solution in the target (i.e., PrC non-preferred) context, and 0% sucrose in the non-target (i.e., PrC preferred) context (Figure 3A). This test is self-executing, and mice were not exposed to any reward or pre-trained during the habituation/PrC sessions. Here, the target and non-target contexts were determined for each mouse during the preconditioning phase, and the output was applied subsequently to the conditioning (learning) sessions (Figure 1E). During the test phase, no reward was presented (retrieval) in both contexts. Analysis of the sCPP% score showed that female mice had robust sensitivity to the absolute sCPP modality, such that target context exploration increased across the conditioning and test sessions. As such, the test sCPP% score for the female cohort was significantly higher than the preconditioning score (*female:* Figure 3B| *t* = −3.47, DF = 9, *p* = 0.007). In contrast, male mice were less sensitive to the absolute sCPP with no significant change in preconditioning and test sCPP% scores (*male:* Figure 3B| *t* = −1.27, DF = 9, *p* = 0.24). Combining the male and female cohorts showed that the group (i.e., M/F) was sensitive to reward learning and context discrimination in the absolute sCPP paradigm (0%/16%). This was mostly driven by female scores (*all:* Figure 3B| *t* = −3.043, DF = 19, *p* = 0.007).

Assessment of sCPP% score during the conditioning and test sessions showed the progression of reward context learning and retrieval of the learned biased context. Progression of sCPP task performance was assessed across experimental sessions for females, males, and male/female cohorts. Among females, the sCPP% score increased empirically from C2 and peaked with the test session (Figure 3C, one-way ANOVA, F = 262.45, *p* < 0.0001). However, there was no significant difference for sCPP% scores when all sessions were compared in a repeated measure One-Way ANOVA (OWA), with Dunnett’s *post-hoc* test. Male mice cohorts attained a comparable proficiency level at C3 (Figure 3D, OWA, F = 111.019, *p* < 0.0001). Together, the sCPP% score for the male/female cohort increased empirically at C2 and significantly at C3 (*p* = 0.02) and Tst (*p* = 0.03) sessions (*versus* the PrC, Figure 3E, F = 295.3, *p* < 0.0001).

### Magnitude sCPP

**FIGURE 4:**
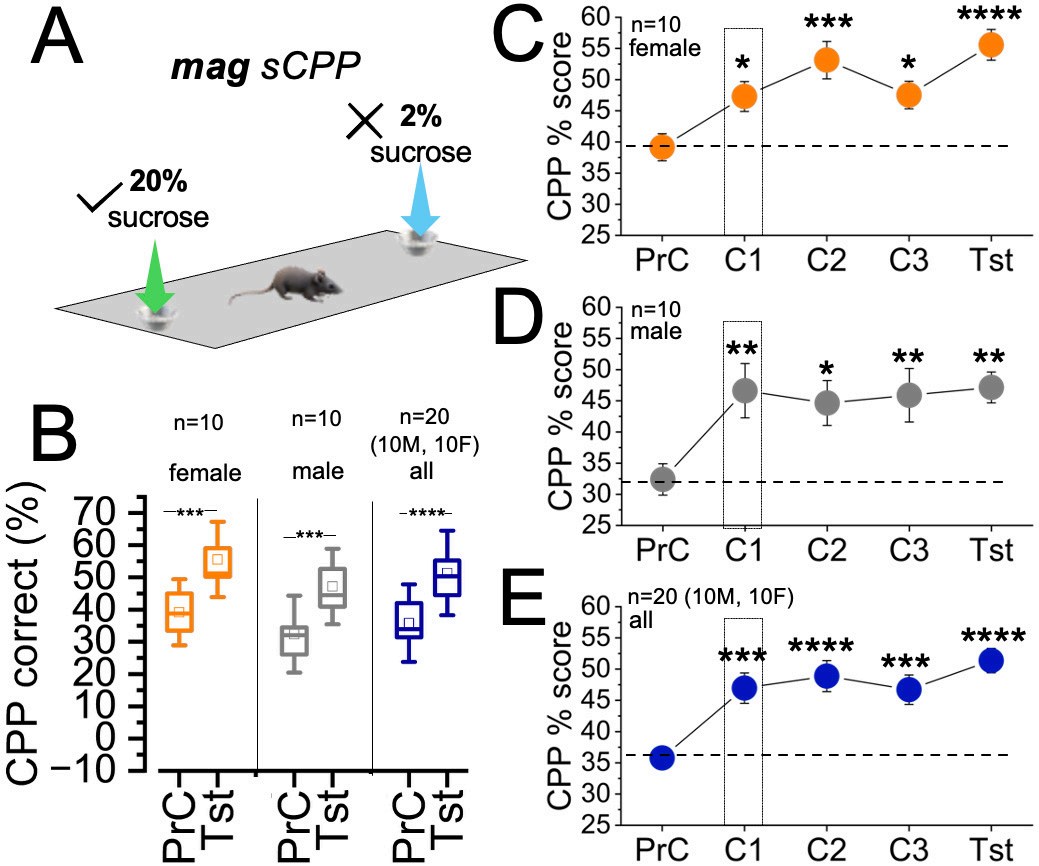
sCPP% score for magnitude sCPP conditioned with 2%:20% sucrose. **(A)** Schematic illustration of *magnitude (mag) sCPP* with biased rewards conditioning. **(B)** Composite graph comparing preconditioning and test sCPP% scores for female (*t* = –5.08, DF = 9, *p* = 6.57e-4), male (*t* = –5.41, DF = 9, *p* = 4.30e-4), and male/female (*t* = –7.55, DF = 19, *p* < 0.0001) cohorts. **(C)** Interval plot comparing female sCPP% scores across sessions (OWA, Dunnett’s *post-hoc* test, F = 1132.348, *p* < 0.0001| *versus PrC*, C1: *p* = 0.048, C2: *p* = 3.4e-4, C3: *p* = 0.041, and Tst: *p* < 0.0001). **(D)** Interval plot comparing male sCPP% scores across sessions (OWA, Dunnett’s *post-hoc* test, F = 285.37, *p* < 0.0001| *versus PrC*, C1: *p* = 0.003, C2: *p* = 0.01, C3: *p* = 0.005, and Tst: *p* = 0.002). **(E)** Interval plot comparing all mice (male/female cohort) sCPP% scores across sessions (OWA, Dunnett’s *post-hoc* test, F = 876.5, *p* < 0.0001| *versus PrC*, C1: *p* = 1.06e-4, C2: *p* < 0.0001, C3: *p* = 1.52e-4, and Tst: *p* < 0.0001). *p < 0.05, **p < 0.01, ***p < 0.001, ****p < 0.0001.

Here, sucrose rewards were presented in both contexts during the conditioning (learning) phase (Figure 4A). A biased discrimination condition was set such that the magnitude of the reward in the PrC non-preferred context (target) was 10 times (20% sucrose) the weight of the PrC preferred context (non-target, 2% sucrose). Female mice showed a significant increase in sCPP% when the test phase was compared with the PrC (*female:* Figure 4B, *t* = −5.08, DF = 9, *p* = 6.57e-4). Similarly, male mice showed robust sensitivity to mag sCPP as the sCPP% for the test was significantly higher than the PrC score (male: Figure 4B, *t* = −5.41, DF = 9, *p* = 4.30e-4). A cohort of male/female mice also showed significantly higher test sCPP% than the PrC score (*all:* Figure 4B, *t* = −7.55, DF = 19, *p* < 0.0001).

Comparison of female mag sCPP% score across PrC, C1-C3, and Tst sessions (OWA RM, Dunnett’s *post hoc*) showed a significant increase in updated place preference from the first conditioning session, C1 (*p* = 0.048). The increased mag sCPP% was sustained for subsequent conditioning sessions C2 (*p* = 3.4e-4) and C3 (*p* = 0.041), and the test session (*p* < 0.0001) (Figure 4C, F = 1132.4, *p* < 0.0001). Similarly, compared to the PrC phase, male mice showed significantly higher *mag sCPP%* scores during C1 (*p* = 0.003), C2 (*p* = 0.01), C3 (*p* = 0.005), and the test session (p = 0.002) (Figure 4D, F = 285.37, p < 0.0001). Together, a combined group of male and female mice showed a significant increase in mag sCPP% score for C1 (*p* = 1.06 e-4), C2 (*p* < 0.0001), C3 (*p* = 1.52 e-4), and the test (*p* < 0.0001) sessions, when compared with the PrC outcomes (Figure 4E, F = 876.5, *p* < 0.0001).

### Learning outcomes for reward magnitude discrimination are stronger than reward detection

**FIGURE 5:**
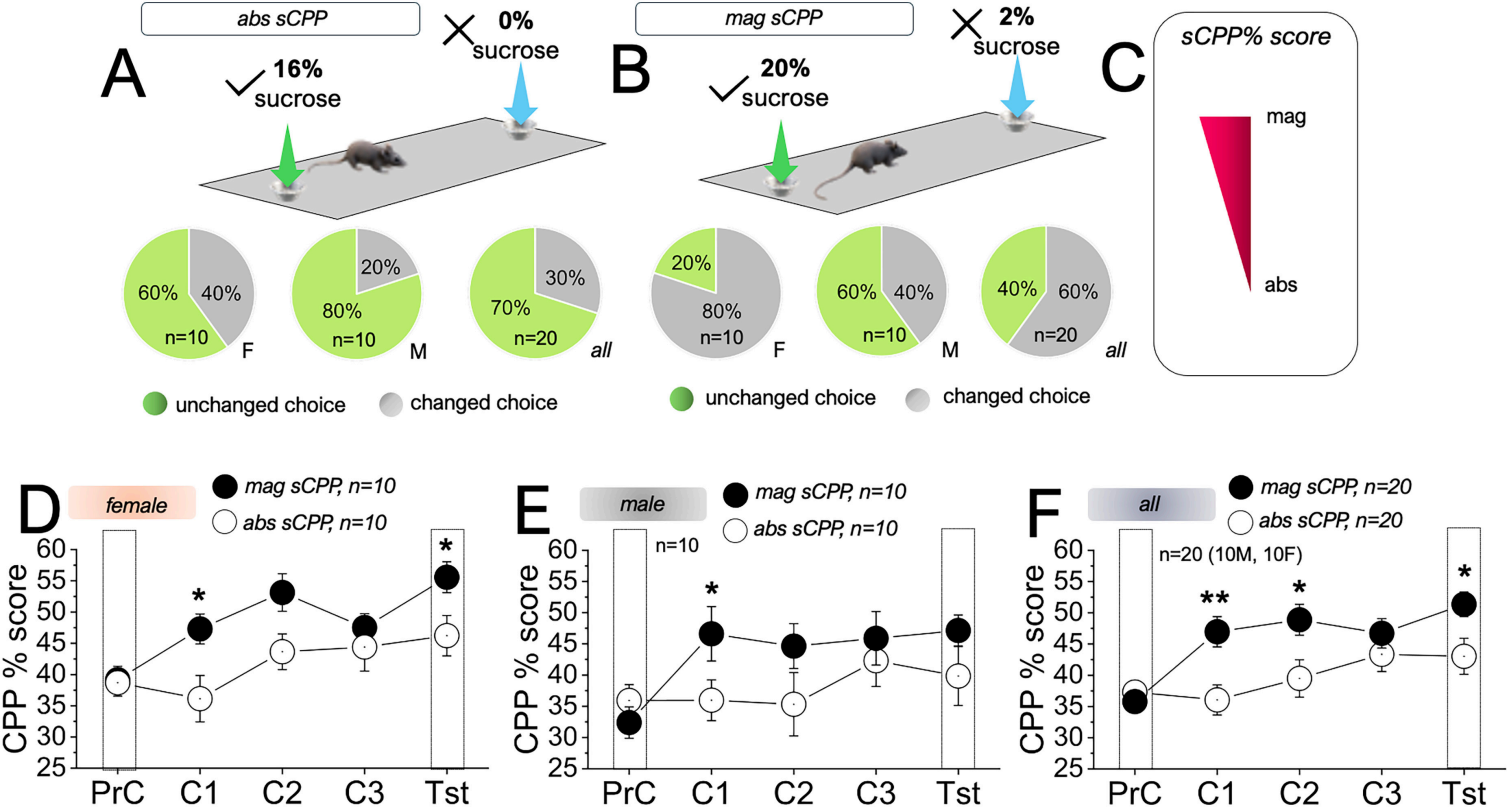
Comparison of absolute and magnitude CPP factors **(A,B)**. Pie charts showing the percentage distribution of mouse sessions with changed preference for absolute (abs) and magnitude (mag) sCPP tasks. **(C)** Schematic illustration of sCPP sensitivity based on modality (*abs or mag*). **(D)** Interval plot comparing abs and mag sCPP% scores for female mice. Significance (*) represents the difference between abs and mag sCPP% scores for the corresponding session (paired *T*-test, C1: *t* = –2.53, DF = 9, *p* = 0.03 and Tst: *t* = –2.42, DF = 9, *p* = 0.038). **(E)** Interval plot comparing abs and mag sCPP% scores for male mice (paired *T*-test, C1: *t* = –2.493, DF = 9, *p* = 0.03). **(F)** Interval plot comparing abs and mag sCPP% scores for the male/female (all) cohort. (paired *T*-test, C1: *t* = –3.65, DF = 19, *p* = 0.002, C2: *t* = –2.47, DF = 19, *p* = 0.02, and Tst: *t* = –2.78, DF = 19, *p* = 0.012). *p < 0.05, **p < 0.01.

In sCPP tests, context learning can be assessed during the conditioning phase. Across three biased conditioning sessions, the progression of contextual preference change can also be determined. Therefore, when the reward is unexpectedly omitted during the test sessions, memory retrieval related to biased conditioning can be further assessed. An increase in sCPP% score during the conditioning and test sessions indicates that the animal explored the target context more than they explored it in the PrC (i.e., sCPP%), as described in Figures 3, 4. For qualitative analysis of task performance, we examined the percentage of mice that recorded a target (Tg) time that is more than the non-target (nTg) exploration time during the test session. This qualitative analysis showed that the female cohort performed empirically better than males for abs sCPP and mag sCPP tasks. Likewise, both male and female cohorts have stronger empirical performance for mag sCPP (2%/20%), in comparison with abs sCPP (0%/16%) tasks. Thus, to assess changed decision during test sessions, the exploration duration of the PrC non-preferred side (Tg) must be greater than the duration in the initially preferred PrC context (nTg). In abs sCPP (Figure 5A), female mice recorded 40% of total changed preference during the test phase, while male mice recorded 20% changed preference. For the combined male/female cohort, there was a 30% (mean) change in preference during the test phase of the abs sCPP task. In comparison, female mice recorded an 80% change in preference during the test phase of mag sCPP tasks, while males had a 40% change in preference (Figure 5B). Together, the male/female cohort showed a 60% (mean) change in preference for the mag sCPP task.

These distribution patterns suggest that learning outcomes for mag sCPP are likely robust compared to abs sCPP in both male and female mice (Figure 5C), and no statistical difference was observed between male and female cohorts. The suspected difference in sensitivity to mag sCPP was further verified statistically by comparing the sCPP scores for the abs and mag experimental sessions. Among female mice, there was no significant difference in preconditioning sCPP% for abs and mag sCPP tasks. This result indicates that sensory cue changes across experiments did not alter preference exploration and performance outcomes (*t* = −0.17, DF = 9, *p* = 0.87, Figure 5D). Interestingly, mag sCPP scores during the first conditioning (C1, *t* = −2.53, DF = 9, *p* = 0.03) and test session (Tst, *t* = −2.42, DF = 9, *p* = 0.038) were significantly higher than abs sCPP scores. There was no significant difference between abs sCPP and mag sCPP scores during C2 (*t* = −2.103, DF = 9, *p* = 0.07) or C3 (*t* = −0.57, DF = 9, *p* = 0.58) (Figure 5D). Male preconditioning abs sCPP and mag sCPP scores were not significantly different (Figure 5E, *t* = 1.35, DF = 9, *p* = 0.21). For the male cohort, the mag sCPP score was significantly higher than abs sCPP during C1 (*t* = −2.49, DF = 9, *p* = 0.03), but not C2 (*t* = −1.49, DF = 9, *p* = 0.17), C3 (*t* = −0.96, DF = 9, *p* = 0.36), or the test (*t* = −1.53, DF = 9, *p* = 0.16) sessions. Combining males and females showed that mag sCPP is more sensitive than abs sCPP. In the learning (conditioning) phase, conditioning with 2%/20% sucrose produced stronger context preference changes when compared with the 0%/16% conditioning (Figure 5F). It is important to note that there was no significant difference in the PrC scores for mag sCPP and abs sCPP (*t* = 0.787, DF = 19, *p* = 0.44). Interestingly, mag sCPP outcomes were significantly higher than abs sCPP scores for C1 (*t* = 3.65, DF = 19, *p* = 0.002) and C2 (*t* = −2.47, DF = 19, *p* = 0.02) but not C3 (*t* = −1.04, DF = 19, *p* = 0.3). Ultimately, the mag sCPP test session score was significantly higher than the abs sCPP outcome (*t* = −2.78, DF = 19, *p* = 0.012).

### Mechanism for dCA1 encoding of context

The behavioral tasks described above showed that mice have a higher sensitivity to reward magnitude discrimination (mag sCPP) compared to reward detection (abs sCPP) (Figures 5D–F). Behavioral sensitivity was derived as sCPP% and represents the threshold of preference change when the task is biased by a reward or a reward of higher magnitude. Given that the hippocampus contains a mix of cells that encode spatial orientation, reward probabilities, and other sensory cues, we propose that dCA1 neurons encode contextual preference at the population level. Within this framework, we determined whether the spatiotemporal coding pattern of dCA1 single unit population that represent an initial contextual preference is updated relative to a physical (behavioral) change in contextual preference. To test this proposition, we determined whether (i) a change in the firing rate, (ii) time of firing relative to context entry, and (iii) peak firing positions predict an initial contextual preference, and subsequently updated preference in sCPP tasks. Lastly, we determined if, and how, dCA1 single units respond to context discrimination paradigms that are driven by absolute and magnitude factors relative to the behavioral task outcomes described above. Spatiotemporal analysis of single units was performed to determine the relative location (i.e., context) where peak firing is attained. Within the hippocampus, reward/context-sensing cells were distinguished from place cells by their lower peak firing rate and change in peak firing position across sCPP task sessions.

#### Absolute sCPP task outcomes during dCA1 single unit recording

**FIGURE 6:**
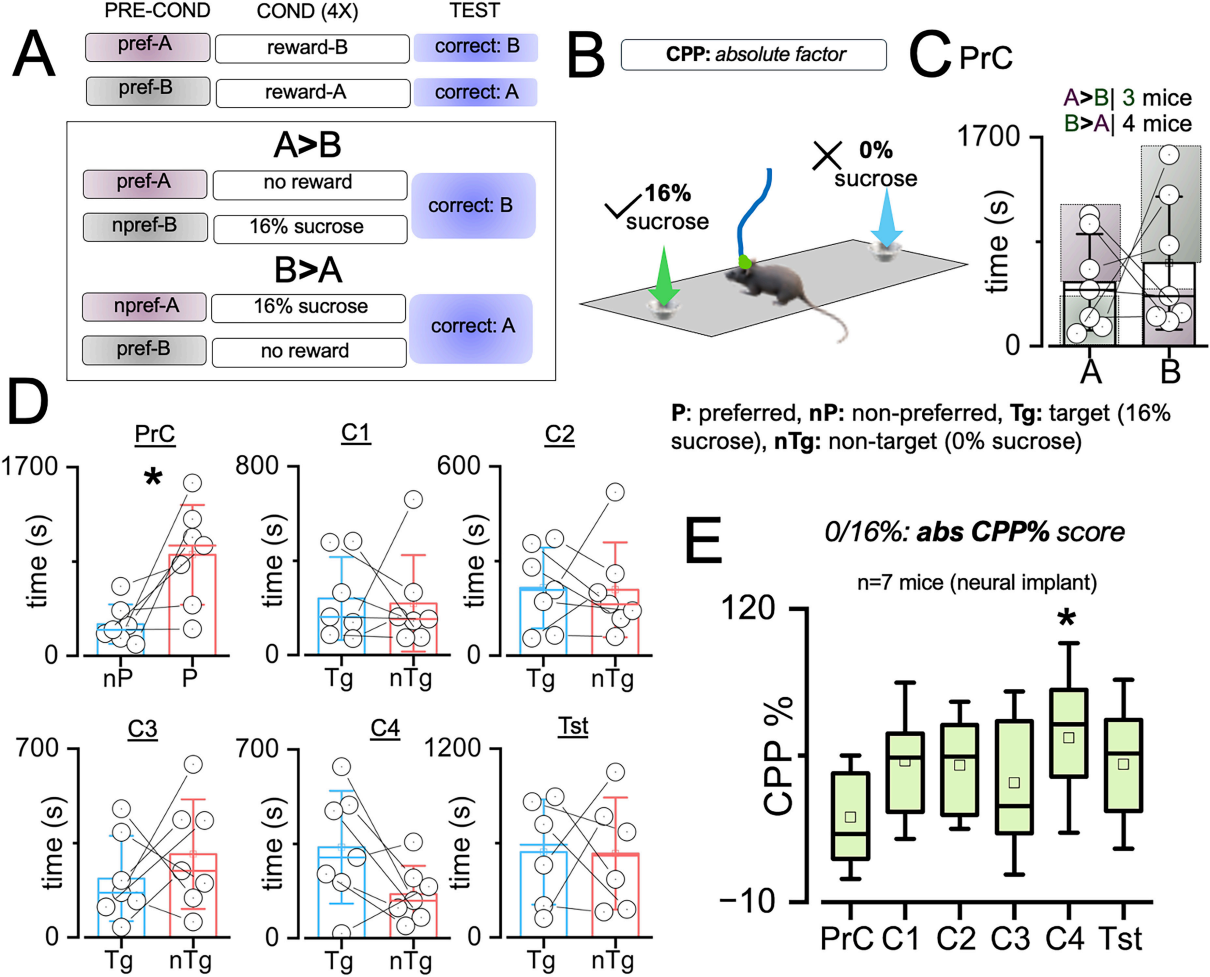
Absolute sCPP outcomes for mice with neural implants. **(A)** Schematic illustration of rewards placement (0%:16%) for mice with context A or B preconditioning preference. **(B)** Schematic illustration of a mouse with neural implants during *absolute (abs) sCPP* task. **(C)** Graph showing time distribution in contexts A and B during the preconditioning phase. *n* = 3 mice preferred context A while *n* = 4 mice preferred context B. **(D)** Graphs comparing exploration time in the preferred and non-preferred contexts during the preconditioning session (paired *T*-test, PrC: *t* = –3.01, DF = 6, *p* = 0.024), then target and non-target contexts for the conditioning (C1: *t* = 0.193, DF = 6, *p* = 0.85, C2: *t* = 0.093, DF = 6, *p* = 0.93, C3: *t* = –0.8153, DF = 6, *p* = 0.45, C4: *t* = 1.9247, DF = 6, *p* = 0.10) and test (paired *T*-test, Tst: *t* = 0.045, DF = 6, *p* = 0.966) sessions. **(E)** Graph comparing abs sCPP% scores across experimental sessions (OWA, Dunnett’s *post-hoc* test, F = 83.68, *p* = 2.62e-4| C1: *p* = 0.33, C2: *p* = 0.23, C3: *p* = 0.73, C4: *p* = 0.047, and Tst: *p* = 0.39). *p < 0.05.

Mice with neural implants performed abs sCPP with four conditioning sessions (Figures 6A,B). After the preconditioning phase (without reward), three and four mice (*n* = 7), respectively, preferred context A and B (Figure 6C). To estimate task performance outcomes, preconditioning outcomes were reorganized such that all context preference (Pr) exploration duration was compared with all non-preference (nPr). The time spent exploring the preferred context was significantly higher than the non-preferred context exploration time (Figure 6D, *t* = −3.013, DF = 6, *p* = 0.024). This serves as the baseline abs sCPP% score, and the non-preference (nPr) was set as the target (Tg) for the subsequent sessions (C1–C4 and Tst). Across conditioning (C1–C4) and Tst sessions, the exploration time was comparable for target (Tg) and non-target (nTg) contexts (*no significance*). Mice with neural implants (Figure 2B) showed an empirical increase in abs sCPP% score when 16% and 0% sucrose were presented at the Tg and nTg contexts, respectively (Figure 6E, F = 83.68, *p* = 2.62e-4). Compared with the pre-conditioning (baseline), there was no significant change in sCPP% during C1-C3, and a significant increase at C4 (Figure 6E, OWA *Dunnett’s post hoc*, *p* = 0.047). Like the observation for mice (male) without implants, there was an empirical increase in abs sCPP% score when the Tst was compared with the PrC session (Figure 6E, F = 83.68, *p* = 2.62e-4).

#### Magnitude sCPP outcomes during dCA1 single unit recording

**FIGURE 7:**
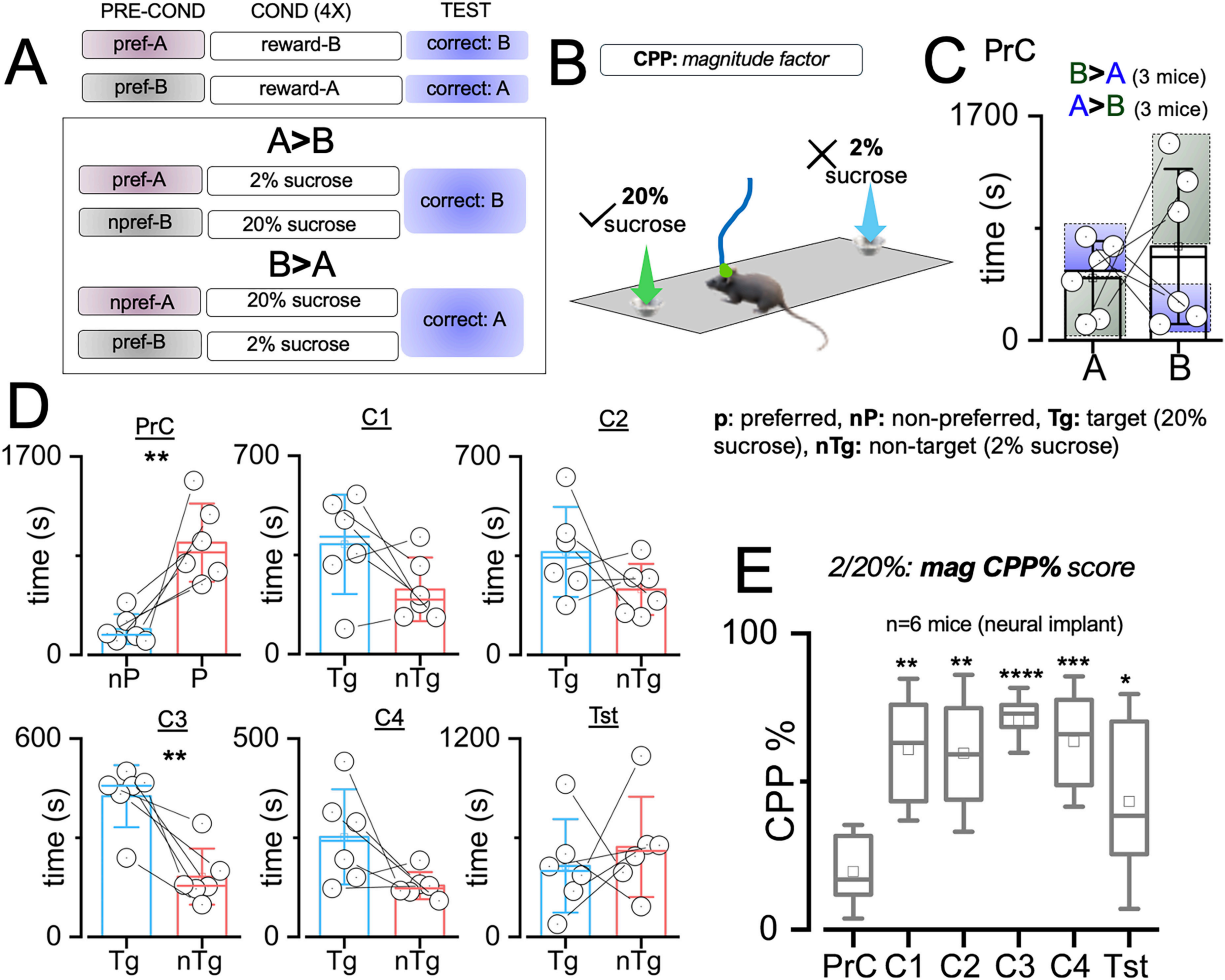
Magnitude sCPP outcomes for mice with neural implants. **(A)** Schematic illustration of rewards placement (2%:20%) for mice with context A or B preconditioning preference. **(B)** Schematic illustration of a mouse with neural implants during the *magnitude (mag) sCPP* task. **(C)** Graph showing the context exploration time (seconds) during pre-conditioning for *n* = 6 mice. Three mice each preferred context A or context B. **(D)** Graphs comparing exploration time in the preferred and non-preferred contexts during the preconditioning session (paired *T*-test, PrC: *t* = –4.6, DF = 5, *p* = 0.0057), then target and non-target contexts for the conditioning (paired *T*-test, C1: *t* = 1.97, DF = 5, *p* = 0.11, C2: *t* = 1.3998, DF = 5, *p* = 0.22, C3: *t* = 6.16, DF = 5, *p* = 0.0016, C4: *t* = 2.15, DF = 5, *p* = 0.084) and test (paired *T*-test, Tst: *t* = –0.63, DF = 5, *p* = 0.57) sessions. **(E)** Graph comparing mag sCPP% score across experimental sessions (OWA, Dunnett’s *post-hoc*, F = 159.6, *p* = 2.26e-4| *versus PrC*, C1: *p* = 0.0016, C2: *p* = 0.0021, C3: *p* < 0.0001, C4: 4.72e-4, and Tst: *p* = 0.0297). *p < 0.05, **p < 0.01, ***p < 0.001, ****p < 0.0001.

The modified CPP task combines a biased conditioning regime with a reward magnitude gradient between two contexts. As such, the preferred context – during preconditioning – was the non-target that contained the lower magnitude reward (2% sucrose). The non-preferred context during preconditioning was used as the target where the higher magnitude reward was presented (20% sucrose) (Figures 7A,B). Analysis of the exploration time for *n* = 6 mice revealed that three mice preferred context A (A > B t secs), while the other three preferred context B (B > A t secs) during the preconditioning session (Figure 7C). Reward placement was reorganized such that the context for PrC preference was set as the “non-target” in the subsequent conditioning phases, while the non-preferred context was set as the “target.” For the PrC session, the non-preferred context exploration was significantly lower than the preferred context duration (Figure 7D, *t* = −4.63, DF = 5, *p* = 0.0057). In subsequent conditioning sessions, exploration time was empirically (C1, C2, C4) or significantly (C3, *t* = 6.159, DF = 5, *p* = 0.0016) reversed, with mice showing affinity for the target with 20% sucrose reward presentation (Figure 7D). Analysis of mag s*CPP% score* showed that reward gradient conditioning drives preference change across the conditioning (C1: *p* = 0.0016, C2: *p* = 0.0021, C3: *p* < 0.0001, and C4: 4.72e-4) and test sessions (Tst: *p* = 0.03); versus PrC (Figure 7E, One-way ANOVA, *Dunnett’s post-hoc test*). This result further confirms that mice have a robust sensitivity to reward magnitude discrimination (Figure 7, F = 159.57, *p* = 2.261e-4) and agrees with the outcomes of the male/female cohort without neural implants (Figure 5).

#### Context-sensing cells in CA1 putative pyramidal single units

**FIGURE 8:**
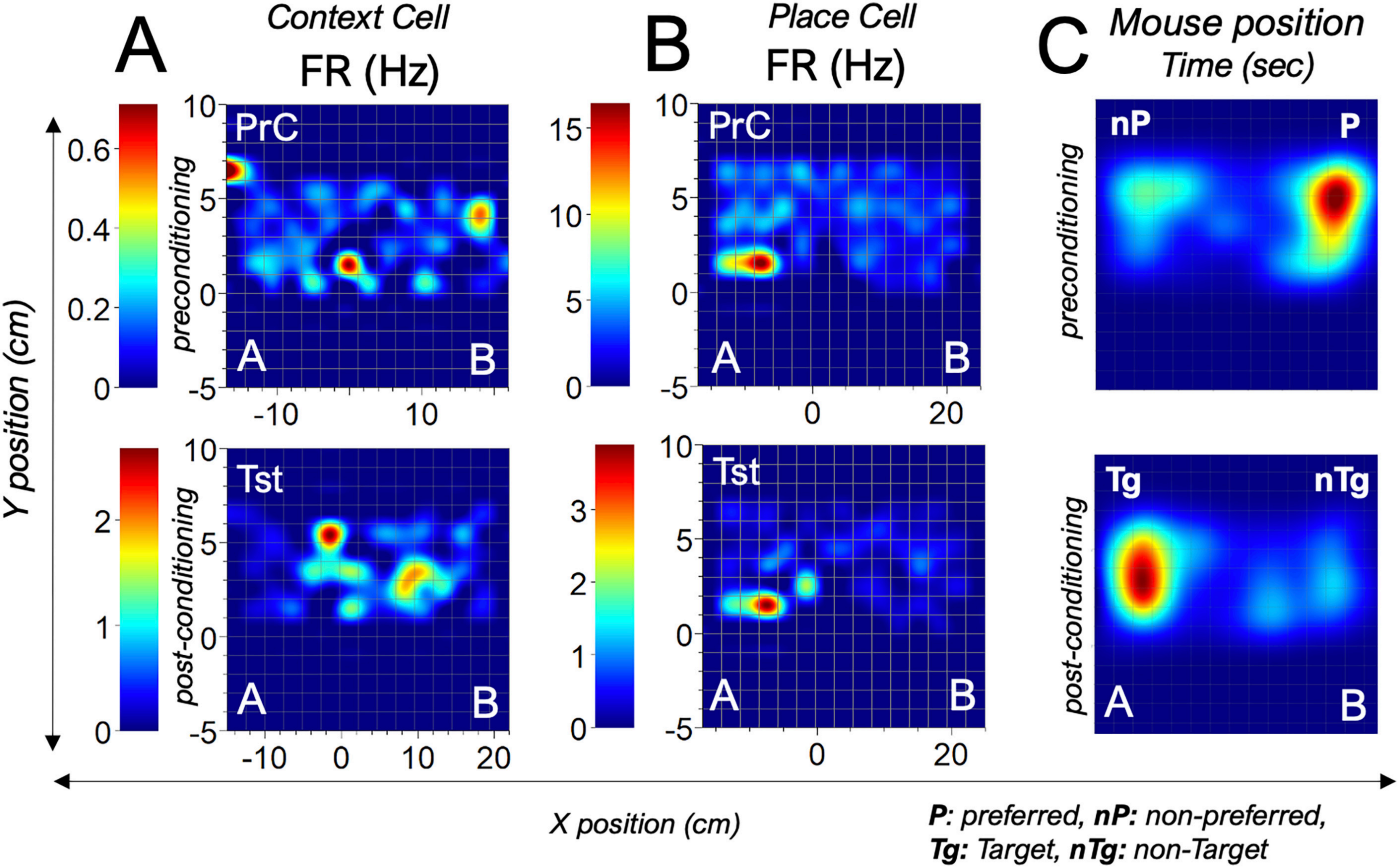
Spatiotemporal heatmaps for putative dCA1 pyramidal neurons showing the peak firing positions of **(A)** a context/reward sensing cell and **(B)** a place cell during the preconditioning and test sessions. **(C)** Heatmap showing the chamber exploration for the subject mouse during the PrC and Tst sessions.

**FIGURE 9:**
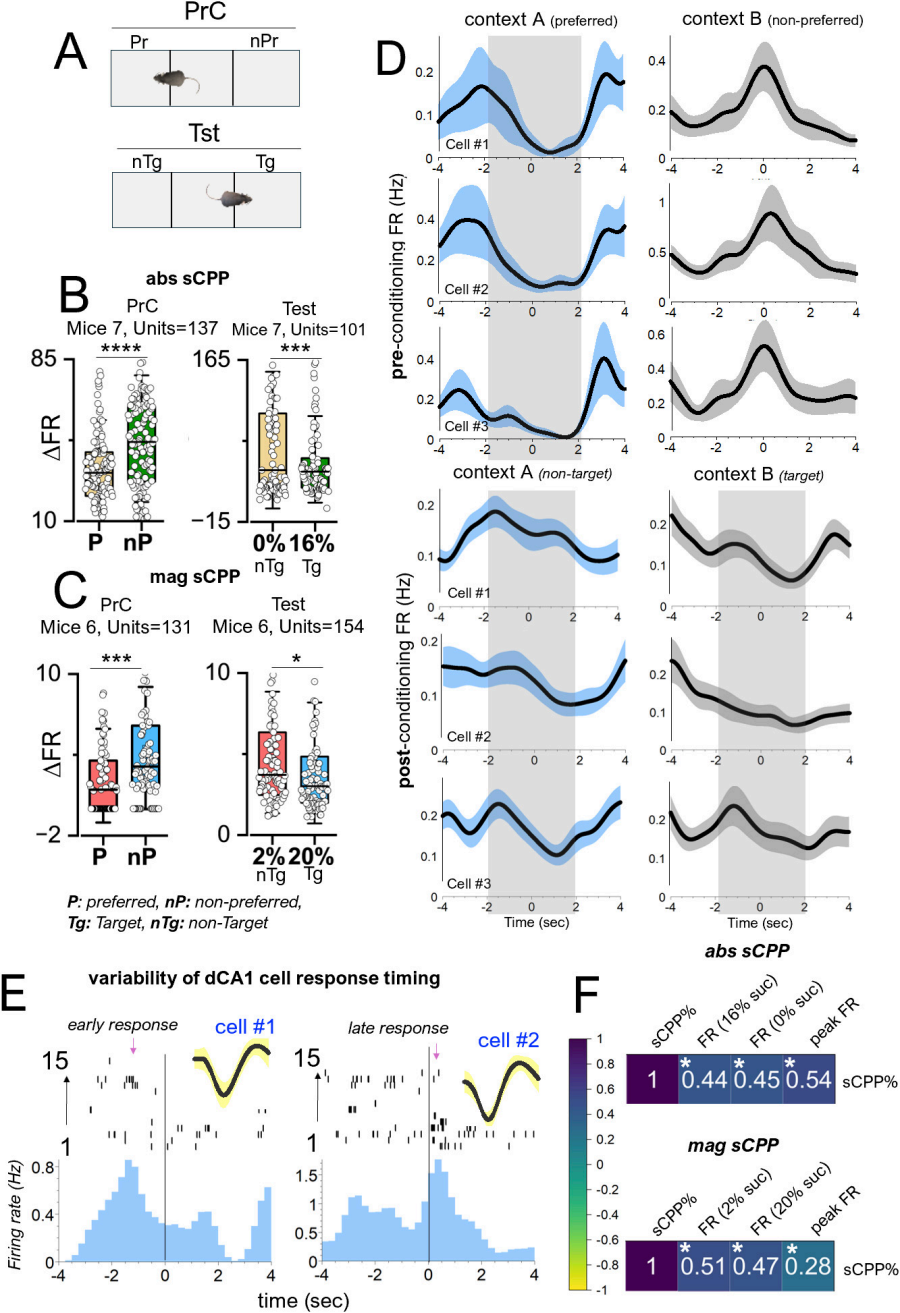
Change in FR of dCA1 cells predicts contextual preference: **(A)** Schematic illustration of context preference during preconditioning and rewards placement in the non-preferred context (nPr) as the target (Tg). **(B)** Change in firing rate (ΔFR) during preconditioning and test phases in the absolute sCPP (*T*-test, PrC: *t* = 5.49, DF = 136, *p* < 0.0001, Tst: *t* = –3.50, DF = 99, *p* = 6.92e-4). **(C)** Change in firing rate (ΔFR) during preconditioning and test phases in the magnitude sCPP (*T*-test, PrC: *t* = –3.48, DF = 91, *p* = 7.83e-4, Tst: *t* = 2.13, DF = 79, *p* = 0.037). **(D)** Perievent firing rate spread for dCA1 putative units during the pre-conditioning and post-conditioning sessions. **(E)** Peristimulus histograms showing typical responsive units relative to zone entry events. **(F)** Correlation plot showing a significant positive relationship between sCPP% and the mean firing rate of dCA1 units during context exploration events. *p < 0.05, ***p < 0.001, ****p < 0.0001.

Spatiotemporal analysis of dCA1 putative pyramidal cell firing rates was used to identify cells that were responsive during context entry events. From this dCA1 population, place cells with fixed firing positions were distinguished from context/reward-sensing cells with changing peak firing positions relative to context preference and reward placement (Figures 8A–C). Context cells in dCA1 putative units have peak positions that are remapped with a change in context preference in an sCPP task driven by reward or reward magnitude (Figure 9). The timing of firing relative to context entry was determined using perievent firing rate (−4 0 4 s). The 0 s mark connotes a TTL time stamp for context entry. Change in firing rate (ΔFR) for context entry was determined by dividing the peak firing rate by the spontaneous firing rate of the PYR cell. Spatiotemporal mapping of the context/reward sensing cells was performed by synchronizing the firing rate of dCA1 neurons and the mouse XY coordinate (cm) in the CPP chamber. In subsequent analysis, the peak firing positions of the reward-sensing cells were compared with the mouse contextual preference across experimental sessions.

#### Putative dCA1 pyramidal cells

After characterizing putative pyramidal cells that were responsive to context and rewards, the following number of cells was used to determine putative unit response across sCPP task sessions. *abs sCPP tasks:* Across experimental sessions, the total number of units sampled in *n* = 7 mice was PrC:137, C1:161, C2:150, C3:172. C4:165, and Tst:101. *mag sCPP tasks:* For *n* = 6 mice, an average of 146 dCA1 putative units were sampled per experimental session (PrC:131, C1:153, C2:158, C3:146, C4:140, and Tst:154).

### dAC1 putative unit firing rate modulation encodes contextual preference

Our results showed striking similarities between dCA1 putative units encoding of two contexts assessed in abs and mag sCPP. Perievent analysis of putative units showed that the dCA1 PYR firing increased during context entries in the PrC and Tst sessions (Figure 9A). Interestingly, analysis of the threshold of firing rate increase, expressed as ΔFR, showed that dCA1 putative units encode context preference. In the PrC, without a reward, dCA1 putative ΔFR indicated a higher threshold that was inversely correlated with the behavioral context preference (Figure 9B). Therefore, in abs sCPP, the ΔFR for the non-preferred was significantly higher than the preferred context entry (*t* = 5.488, DF = 136, *p* < 0.0001). Similarly, in the mag sCPP (Figure 9C), the ΔFR for the non-preferred was significantly higher than the preferred context entry (*t* = −3.475, DF = 91, *p* = 7.83e-4) during the PrC session (no rewards). These results suggest that the dCA1 putative units are context-sensing and can predict spatial preference through ΔFR directionality, without a reward. It follows that the ΔFR directionality of these cells is further updated when the contextual preference is reversed by biased reward contingencies. Behavioral test outcomes showed that these mice changed their contextual preference when biased with a reward (abs sCPP, Figure 6E) or rewards magnitude (mag sCPP, Figure 7E). During the test session (Figures 9B,C), the ΔFR for entry into 0% or 2% non-target contexts was significantly higher than 16% (abs sCPP, *t* = −3.50, DF = 99, *p* = 6.92e-4) or 20% (mag sCPP, *t* = 2.13, DF = 79, *p* = 0.037) target contexts, respectively. These results showed that reward/context detection increases the firing rate in dCA1 single units more robustly at sites that are not spatially preferred during the PrC or targeted during the test sessions. In support of this proposition, perivent FR curves for three cells are shown to demonstrate the tuning of dCA1 rewards cells during context entry events (Figure 9D). Peristimulus histograms further demonstrate the variability of cell response around context entry events during sCPP tasks, with some cells responding shortly before, and others responding during the entry (Figure 9E). In support of the proposition that the firing of these cells is linked to the behavioral outcomes, correlation plots show a significant positive relationship between sCPP% and the mean firing rate of dCA1 units during context exploration events (Figure 9F).

**FIGURE 10:**
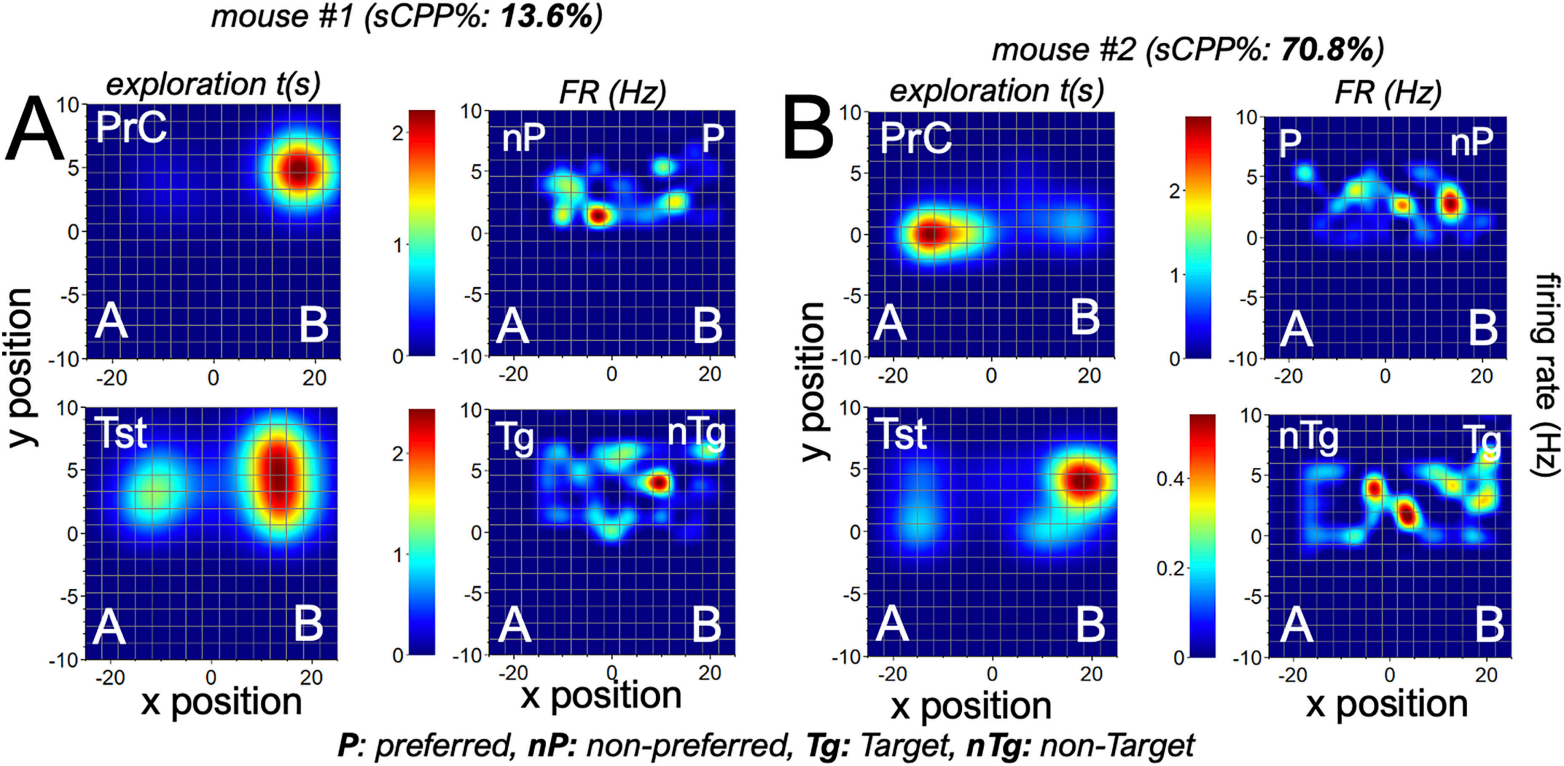
Spatiotemporal remapping of dCA1 cells in sCPP task: **(A,B)** Representative heat maps demonstrating mouse chamber exploration (left) and firing positions of a putative dCA1 neuron (right). Cells were sampled from the dCA1 of mice with low (*mouse 1*, 13.6%) or high (*mouse 2*, 70.8%) sCPP% score. Both mice had opposite but strong contextual preferences in the preconditioning session (*mouse 1:* B and *mouse 2:* A). In the preconditioning sessions, the peak firing position of these cells occurred around the non-preferred context, thus opposite to the animal’s contextual preference. After the conditioning phases, biased reward presentation altered the value of the context and exploration preference (*mouse 2:* B). A putative dCA1 context-linked neuron tuned its peak FR position to update the preference change in high-performing mouse 2 but not in the low-performing mouse 1.

To verify this outcome, we examined the spatiotemporal map for typical putative units in the dCA1 of two mice with *high* or *low sCPP% test scores*. Also, these mice had different PrC preferences. Mouse 1 had a low sCPP score (Figure 10A, 13.6%), while Mouse 2 had a higher sCPP score (Figure 10B, 70.8%). During the PrC session, Mouse 1 preferred context B, while Mouse 2 preferred context A. Spatiotemporal heat maps showed that the peak firing position of a neuron occurred in the non-preferred PrC context for both *Mouse 1* and *Mouse 2*. However, during the test session, the peak firing position occurred around non-target contexts or away from target contexts. The mouse with a high-biased sCPP% score had more defined or multiple peak firing positions toward the non-target compared with the low-scoring mouse, which had a less defined or single peak position. Based on these observations, we deduced that the spatiotemporal change in peak FR of these cells encodes context preference and is subsequently updated after biased conditioning. To verify the relationship between the dCA1 reward cell encoding and the behavioral outcomes in sCPP tasks, we determined whether the sCPP% score has correlations with the firing rate during target events in the task chamber. To achieve this, the mean of FR for reward cells was determined per mouse and compared against the sCPP% score for each mouse during the PrC, C1–C4, and Tst sessions. This correlation was derived for abs and mag sCPP sessions. Our results demonstrate a positive correlation between the sCPP% outcomes and the firing rate during context entry events, irrespective of the associated rewards value. In support of this observation, a positive correlation was also observed when the peak firing rate in spatiotemporal mapping was compared against the sCPP% score (Figure 9F). Therefore, a change in firing rate (ΔFR) during target events depicts the normalized firing threshold that encodes the rewards paradigms associated with the contexts.

### Spatiotemporal Δpeak FR encodes reward detection and magnitude-driven tasks

**FIGURE 11:**
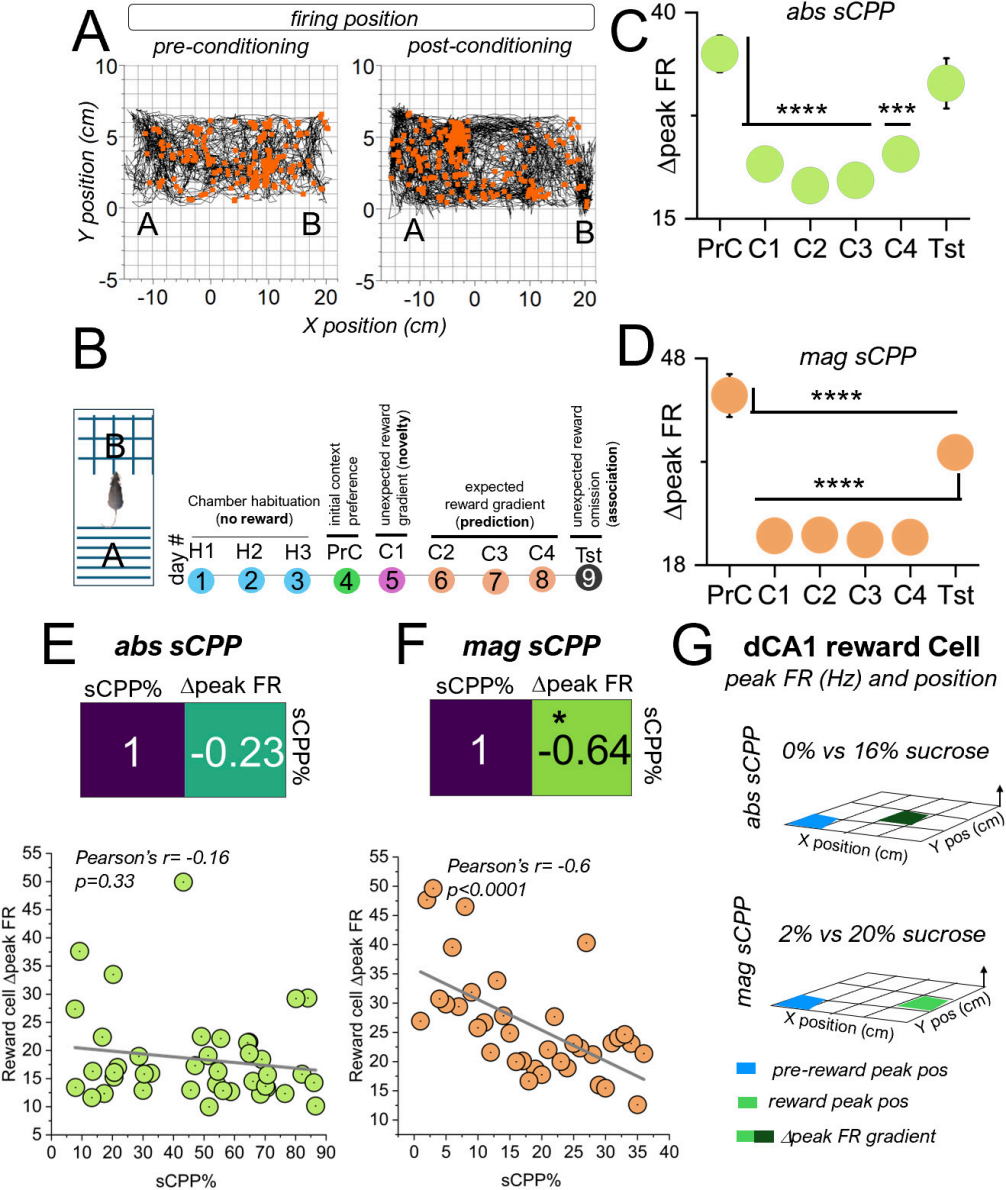
Spatiotemporal coding pattern in dCA1 pyramidal single unit population during rewards learning and context discrimination progression. **(A)** Sample firing position of putative units during the preconditioning and test phases. **(B)** Illustration of rewards task learning and context discrimination phases of sCPP tasks. **(C,D)** Graph comparing the dCA1 Δpeak FR across **(C)** absolute sCPP and **(D)** magnitude sCPP experimental sessions (OWA, Dunnett’s *post-hoc* test, *Abs sCPP*, F = 835.88, *p* < 0.0001| versus PrC, C1: *p* < 0.0001, C2: *p* < 0.0001, C3: *p* < 0.0001, C4: *p* = 05.52e-4 and Tst: *p* = 0.68, and *Mag sCPP*, F = 2254.7, *p* < 0.0001| C1-C4 and Tst, *p* < 0.0001). **(E,F)** Correlation analysis (top) and linear regression plot (with fitting, bottom) show an inverse relationship between sCPP% outcomes and the Δpeak FR (Pearson’s r). The negative correlation was not significant for abs sCPP and was significant (*p* < 0.05) for the mag sCPP sessions. **(G)** Schematic illustration of rewards cell remapping relative to Δpeak FR and sCPP modality. *p < 0.05, ***p < 0.001, ****p < 0.0001.

Spatiotemporal analysis of the peak firing rate and the approximate position in which peak firing was attained showed that the Δpeak FR encodes reward detection during the conditioning phase, and reward omission during the test phase (Figure 11A). For this analysis, we considered the various stages of the sCPP task as follows. The PrC phase established the natural spatial preference of a mouse without a reward present. During the first conditioning phase (C1), rewards(s) were unexpectedly encountered. From C2 to C4, the rewards was expected and predicted (learning). Lastly, rewards were unexpectedly omitted during the test phase (retrieval) (Figure 11B). For both abs (OWA, F = 835.88, *p* < 0.0001) and mag sCPP (OWA, F = 2254.7, *p* < 0.0001) tasks, the Δpeak FR decreased significantly during the conditioning phases (C1–C4) when a reward was presented. Interestingly, when the rewards were unexpectedly omitted during the test phase, the Δpeak FR increased for dCA1 single units. Since the decrease in Δpeak FR occurred during both abs and mag sCCP, we inferred that this is an encoding mechanism for reward detection that does not discriminate between abs and mag modalities. Interestingly, for the abs sCPP test session Δpeak FR was comparable with conditioning Δpeak FR (Figure 11C, *p* = 0.68, OWA RM, Dunnett’s *post hoc*). Conversely, mag sCPP test session Δpeak FR was significantly higher than the conditioning Δpeak FR (Figure 11D, *p* < 0.0001). In support of this outcome, correlation analysis revealed an inverse relationship between the sCPP% score and the Δpeak FR. As such, in the abs sCPP sessions, the negative correlation (Figure 11E, r = −0.23, *p* > 0.05) or linear regression (*r* = −0.16, *p* = 0.33) depicting the link between sCPP% scores (Figure 6E) and Δpeak FR (Figure 11C) were not significant. Conversely, in mag sCPP tasks (Figure 11F), both the negative correlation (*r* = −0.64, *p* < 0.05) and linear regression (*r* = −0.61, *p* < 0.0001) between sCPP% (Figure 7E) and the Δpeak FR (Figure 11D) were significant. Together, these results suggest that dCA1 single units encode reward detection and may discriminate between absolute and magnitude context discrimination tasks through Δpeak FR modulation (Figure 11G).

## Discussion

Detecting novelty and associating it with context guides the brain to rank environmental stimuli (Adams et al., 2012; Anderson, 2019; Coley et al., 2021; Dalenberg et al., 2018). The neural circuitry between the mesocorticolimbic and cognitive pathways continuously updates the ranked system by assigning valence and tuning the weight of the valence (Orsini and Simon, 2020; Park and Moghaddam, 2017). Results of this study showed that dCA1 single units are “context-sensing” and encode contextual preference. Although the population consists of a variety of cells, the overall timing and threshold of firing rate change during context entry events underscores the initial place preference, and subsequent change in preference when the behavior of the animal is biased with reward cues. It follows that the ΔFR threshold was inversely correlated with exploration and contextual preference. *Here, we deduced that the spatiotemporal pattern of ΔFR of dCA1 single units represents a flexible schema of a current behavioral preference and is updated in real time when behavioral (contextual) preference is reversed by reward contingencies.* In support of this proposition, the ΔFR of context-sensing dCA1 single units is tuned by both simple reward detection (0%/16%) and more complex reward magnitude discrimination (2%/20%) cues. Thus, *post hoc* analysis of ΔFR predicts behavioral outcome such that entry into contexts with no reward (0%, *abs sCPP*) or low magnitude reward (2%, *mag sCPP*) elicited significantly higher ΔFR scores. Together, these results showed that ΔFR directionality is predictive of contextual preference without a reward bias and is updated when contextual preference is reversed by biased conditioning. Analysis of behavioral outcomes for mice without (*n* = 20) and with neural implants (*n* = 7 and *n* = 6) showed that reward magnitude discrimination produces a stronger behavioral response than reward detection. Furthermore, spatiotemporal mapping of dCA1 single units during these tasks revealed that the Δpeak FR encodes reward encounters during the conditioning phase. When the reward was unexpectedly omitted, the Δpeak FR during the mag sCPP test phase was significantly higher than the associated reward encounters (conditioning). Conversely, in reward detection tasks (abs sCPP), there was no significant difference between Δpeak FR scores for the conditioning and Tst session. Thus, the memory trace for reward magnitude discrimination is robust when compared with reward detection.

Traditionally, the CPP task was developed to test the effects of drugs on place preference, such that animals avoid contexts linked to drugs, such as lithium, with sickening effects, and tend to prefer contexts with drugs – nicotine, cocaine, methamphetamines – of addiction (Napier et al., 2013; Şorodoc et al., 2022; Scherma et al., 2021; Huston et al., 2013; García Pardo et al., 2017). The CPP tests can read out spatial contextualization, reward motivation, and executive behavior (decision). In the current study, an affinity for sucrose consumption – after fasting – was preferred for a positive valence stimulus (i.e., reward) since animals have access to these in their natural habitats (Eikelboom et al., 2022; Ackroff et al., 1993; Bradshaw, 2023). Thus, by setting a biased, self-executing paradigm, mice were trained by conditioning and learned to change an individualized ranked context choice that was predetermined per mouse during the preconditioning phase. Here, each set of mice performed the task when the sCPP was conditioned with an absolute factor (0%/16%) and 2 weeks later when the sCPP was conditioned with a magnitude factor (2%/20%). Contextual sensory cues were counterbalanced between sCPP modalities.

### Differential behavioral response to abs and mag sCPP tasks

Previous studies have shown that sex-linked differences in reward-seeking behavior and reward-oriented learning may be constrained by the type of reward (Chowdhury et al., 2019; Xie et al., 2019, Barrett et al., 2020). A previous study also highlights sex-linked differences in sucrose reward affinity in rats, with female rats consuming more sucrose and showing more sensitivity in operant tasks (Grimm et al., 2022). Underlying differences in neural response patterns – especially in the mesocorticolimbic pathway – can further elucidate some of the sex-linked differences in reward preference (Rivera-Garcia et al., 2020; Godfrey and Borgland, 2020; Castro-Zavala et al., 2020) and other adaptive phenotypes – notably stress (Zhang et al., 2018; Douma and de Kloet, 2020). Although drugs of addiction will likely elicit stronger responses – compared to sucrose – in CPP tests, the sucrose reward paradigm is more ethologically relevant in dissecting neural circuits associated with normal foraging behavior. Furthermore, in a biased sCPP, learning can be tracked temporally across the conditioning sessions to deduce a graded outcome for the change in contextual preference.

Our results showed that under similar conditions, cohorts of mice have different sensitivity to *abs sCPP* and *mag sCPP*. In addition, female mice showed a stronger response than males during reward detection and reward magnitude discrimination. Specifically, 40% (abs) or 80% (mag) of female mice and 20% (abs) or 40% (mag) of male mice showed a change in context preference (time sec) during the test sessions (versus PrC). Mice also had a higher sensitivity to mag sCPP in comparison with abs sCPP. Analysis of *sCPP%* scores across experimental sessions revealed an early onset context preference change for mag sCPP in comparison with *abs sCPP*. Here, mag sCPP scores were significantly higher than the abs sCPP% score across conditioning and test sessions.

### dCA1 putative units sense contextual preference in sCPP tasks

The concept of novelty or valence detection is not all or none. It includes complex computation across multiple circuits that control fear, risk value, stress, and executive cognitive functions, including the prefrontal cortex, lateral habenula, and amygdala (Cooper et al., 2006; Kong and Zweifel, 2021; Michel et al., 2024; Zhang and Li, 2018). Nonetheless, the initial computation by the hippocampal-mesocorticolimbic axis is pertinent for the initial risk/value and position assessment. Spatial learning is also a major function of the hippocampus; as such, the location of novel information (e.g., space, object, food) is key to the learning dynamics. Thus, to determine how the CA1 encodes spatial location preference, the presence or absence of rewards and the assigned value of contexts are defining factors. The sucrose conditioned place preference (sCPP) test permits the detection of CA1 spiking events when a mouse enters a contextual location that is preferred compared to one that is not preferred. In addition, we used this test to examine dCA1 single-unit spatiotemporal patterns when the contextual preferences are subsequently biased by a reward or a reward of higher value.

Previous studies have shown that the dorsal CA1 (dCA1) putative units encode spatial location, sensory cues, and rewards (Gauthier and Tank, 2018). Furthermore, there is significant evidence that introducing a reward into the context alters the activity of dCA1 neurons both for its sensory and reward values (Gauthier and Tank, 2018; Russo et al., 2024; Valenti et al., 2018). Recent work demonstrates that dCA1 single units are more heterogeneous than initially thought. Specialized reward-predicting cells encode reward by firing around target locations in shifting reward contingencies. These cells are distinguishable from place cells and remap with the change in reward location (Gauthier and Tank, 2018). Given that the firing patterns of reward cells and the spatial location of their peak firing are goal-directed, the question arises about “how” dCA1 single units encode place preference tasks with a simple goal of obtaining a reward versus a complex goal of discriminating between two reward weights. To separate the effect of sensory cues from reward cues, animals performed four habituation sessions and a preconditioning session during which the animals’ initial contextual preference gradient was determined.

The use of sucrose rewards in these tasks provides ethologically relevant experimental scenarios such that the preconditioning session recapitulates baseline discrimination between two contexts that are not associated with a reward (Ackroff et al., 1993; Adams et al., 2012; Anderson, 2019; Eikelboom et al., 2022). Presentation of reward in a biased modality during the first conditioning session models unexpected reward presentation (reward novelty) at the target and omission or lower value at the non-target. Presenting rewards during the subsequent conditioning sessions creates a premise for assessing reward prediction at the target and omission or lower value at the non-target. Lastly, the test session models an unexpected reward omission at the target. Thus, analyzing the encoding patterns in dCA1 pyramidal neuron population creates a premise for determining how the firing rate of the context-sensing single units computes contextual preference relative to reward contingencies. The results of this study support previous findings that distinguish reward and place cells (Gauthier and Tank, 2018; Jarzebowski et al., 2022). Given that “rewards” are predicted at “locations,” it is logical to speculate that overlap can occur between place and reward fields.

In the dCA1 neuron population, reward cells were responsive to the presentation of rewards or the probability of a reward. Conversely, place cells had fixed firing positions. Our results showed that dCA1 single units use a similar mechanism to track spatial preference (without rewards) and reward-driven contextual preference. Therefore, the ΔFR of these context-sensing single units reflects behavioral task outcomes during the preconditioning phase when the contexts are not associated with a reward. However, when the contexts are biased with a reward, the ΔFR of dCA1 context-sensing single units tracks the updated preference. Although context entry events are generally associated with an increase in FR, our results showed that the less preferred contexts during PrC, or contexts with lower values, have a higher firing rate increase (i.e., ΔFR). These results agree with previous observations that reward cells are more sensitive to unexpected omissions than reward encounters (Gauthier and Tank, 2018). Therefore, when a reward is biased against the animal’s spatial preference, the ΔFR for the non-target (PrC preferred, low value) increased significantly in comparison with the target (PrC non-preferred, high value). This change in ΔFR directionality suggests that the dCA1 population schema is updated with contextual preference.

### Encoding dynamics in dCA1 context-sensing single units

Given that the ΔFR of the dCA1 single units encodes spatial preference without a physical reward, the question arises whether these cells are primarily responsive to space or reward. This question is complex since the perception of a preferred space can also be rewarding. Hence, for the initial (PrC) preference, the ΔFR for the non-preferred space was significantly higher than the preferred space. When the PrC preference is biased by a reward during conditioning sessions, we showed that the ΔFR schema is updated in the dCA1 single unit population. Specifically, in the conditioning phase, reward was presented in the PrC non-preferred side, thus increasing its value over the PrC preferred context. Following the biased reward association, ΔFR increased significantly for the conditioning non-target (PrC preferred), which is the new lower value context. Conversely, the ΔFR decreased for the conditioning target (PrC non-preferred), which is the new high-value context.

To determine if the dCA1 context schema is driven by context or reward, we compared the outcomes for abs and mag sCPP tasks. In abs sCPP, one context was biased by a reward, while the other had no reward, while in mag sCPP, both contexts had a reward. Our results showed that these cells encode contextual preference and detect reward paradigms (*abs* versus *mag*). We also showed that contextual preference was encoded as single units ΔFR tuning. As such, spatiotemporal dynamics for reward contingencies were mapped in single-unit populations as Δpeak FR. Because 0%/16% and 2%/20% were encoded by a similar ΔFR pattern, we deduced that the ΔFR primarily represents place preference and not the reward contingency. In support of this proposition, the ΔFR for lower value contexts (0% or 2%) context entry events was significantly higher than 16% or 20% sucrose contexts. This is similar to the ΔFR pattern observed during PrC, where the non-preferred context entry elicited a higher ΔFR score than the preferred context entry. Based on these results, the ΔFR likely encodes contextual preference, and such encoding patterns are not dependent on reward contingency. As such, the ΔFR directionality, which is inversely related to the contextual preference, was similar for the pre-conditioning and post-conditioning sessions of sCPP tasks.

Spatiotemporal analysis of Δpeak FR across tasks showed that the dCA1 single unit population detects reward in context discrimination tasks. As such, the Δpeak FR for dCA1 neural single unit decreased during the conditioning phases (C1–C4) for both abs sCPP and mag sCPP tasks, compared with the pre-conditioning score. Interestingly, when the reward was omitted in the test phase of mag sCPP, the Δpeak FR was significantly higher than in rewards encounters (C1–C4). Conversely, in abs sCPP tasks, there was no significant difference in the Δpeak FR for reward encounters and subsequent omissions. These results showed that introducing reward into the chamber reduced the Δpeak FR of dCA1 single units and was reversed when the reward was omitted. Furthermore, Δpeak FR for reward magnitude (mag sCPP), and not reward detection (abs sCPP), significantly discriminates encounters and omissions.

#### Spatiotemporal maps of dCA1 single units are driven by behavioral outcomes

Previous studies demonstrate that neural encoding in the hippocampus is predictive and has robust sensitivity to rewards (Chen et al., 2024; Dupret et al., 2010; Lee et al., 2006; Miller et al., 2023; Stachenfeld et al., 2017). In support of this proposition, we showed that the ΔFR and the timing of peak occurrence are initially predictive of spatial preference without a reward associated with the space. Furthermore, when reward is introduced in sCPP tasks, the spatiotemporal map for hippocampal cells was updated. Here, the ΔFR increased significantly at sites with omitted rewards (abs sCPP) or rewards of lower magnitude (mag sCPP). Because of this remapping, the ΔFR at the initial non-preference during preconditioning was higher than the ΔFR at the initial preference. Furthermore, after biased conditioning regimes, the ΔFR at the post-conditioning target (PrC non-preference), which is now associated with reward, was lower than the post-conditioning non-target (PrC preference) without reward. These results agree with the previous studies, which demonstrate remapping and a change in the timing of peak firing of reward cells in long-term reward tasks (Yaghoubi et al., 2026). Similarly, our results also show that the activation pattern of these cells is tuned by behavior, such that mice with higher sCPP scores show distinct and robust ΔFR compared with mice with lower sCPP scores.

#### Spatiotemporal maps of reward cells can encode location preference

There is consensus that reward cells are remapped based on reward prediction and that this remapping is linearly linked to behavior (Dupret et al., 2010; Lee et al., 2006, Stachenfeld et al., 2017; Yaghoubi et al., 2026). Here, we showed that the flexible schema represented by dCA1 single unit populations encodes spatial (location) preference observed in behavioral tasks, and ultimately reflects contextual preference driven by biased reward encounters. Since spatial novelty is rewarding, we inferred that the observed encoding mechanism may apply to the spatiotemporal map of “where (location)” or “what (sucrose reward)” is preferred. During the two separate preconditioning sessions (abs, then mag sCPP) without rewards (3 weeks apart), mice explored and showed a preference, which may be similar or different over time and with the presentation of different tactile, visual, and odorant cues. Our results showed that the ΔFR was higher for the non-preferred side when compared with the preferred side. These results suggest that the proposed encoding mechanism and spatiotemporal maps are strongly linked to contextual preference and can be remapped based on behavioral sensitivity to rewards.

#### Hippocampal encoding of reward detection (abs) and reward comparison (mag) tasks

Given that the spatiotemporal remapping described here elucidates a continuum between location preference and subsequent reward bias, we next determined whether the observed spatiotemporal map distinguished between the preconditioning (no reward), conditioning (reward), and post-conditioning (test, omitted reward) phases. Spatiotemporal analysis across experimental sessions revealed that these cells are more tuned by reward preference rather than by location preference. As such, the Δpeak FR during the conditioning sessions with rewards was significantly lower than the preconditioning scores. These suggests a robust reward sensitivity for these cells. Interestingly, when the reward was omitted subsequently, the Δpeak FR distinguished between abs and mag sCPP modalities. Here, the Δpeak FR for postconditioning mag sCPP was significantly higher than the conditioning peak FR. Conversely, the Δpeak FR for the post-conditioning abs sCPP was not significantly different from the conditioning scores. Thus, there is a stronger sensitivity to reward comparison than reward detection. Furthermore, a stronger linear relationship was observed the Δpeak FR and mag sCPP than for abs sCPP. These results support the proposition that these cells are tuned behaviorally. Furthermore, we showed that the tuning is likely proportional to the sensitivity of the behavioral task. As such, the negative correlation between the Δpeak FR and sCPP% score was significant during the mag sCPP and not the abs sCPP task. The enhanced behavioral sensitivity to mag sCPP tasks can be attributed to the stronger correlation between the Δpeak FR and sCPP% outcomes. Since the cells are remapped spatiotemporally, a stronger behavioral modulation was observed during the mag sCPP, as shown by a significant suppression of Δpeak FR during the conditioning when compared with the preconditioning and post-conditioning phases. Conversely, in the abs sCPP with weak behavioral/Δpeak FR correlation, there was a lower behavioral sensitivity.

## Conclusion

We conclude that dCA1 context-sensing single units encode context preference through a ΔFR schema that is updated with preference changes induced by reward contingencies. We also showed that the Δpeak FR within the spatiotemporal map encodes reward encounters and omissions, with magnitude comparison being more robust than reward detection.

## Data Availability

The raw data supporting the conclusions of this article will be made available by the authors, without undue reservation.
